# COVID-19 and misinformation: how an infodemic fuelled the prominence of vitamin D

**DOI:** 10.1017/S0007114520002950

**Published:** 2020-07-27

**Authors:** Joshua Henrina, Michael Anthonius Lim, Raymond Pranata

**Affiliations:** 1 Siloam Heart Institute, Siloam Hospitals Kebon Jeruk, Jakarta, Indonesia; 2 Faculty of Medicine, Universitas Pelita Harapan, Tangerang, Indonesia; 3 Faculty of Medicine, Universitas Pelita Harapan, Tangerang, Indonesia

We read with great interest of a preprint paper^(1)^ allegedly written by an Indonesian author entitled ‘Patterns of COVID-19 Mortality and Vitamin D: An Indonesian Study’, which has taken the Internet by storm. According to the PlumX Metrics^(2)^, this article has been used more than 100 000 times, downloaded more than 17 000 times and mentioned in social media over 8000 times. Through Google Scholar^(3)^, we found that this article has been cited nine times, six of which are indexed in PubMed. We also found an article in the *British Medical Journal* (*BMJ*)^(4)^ that cited this article, which was not detected by Google Scholar, through a conventional Google search. The manuscript has been cited in COVID-19 rapid evidence summary: vitamin D for COVID-19, an evidence review by National Institute of Health and Care Excellence (NICE)^(5)^. Thus, we believe that the impact on scholarly fields is far more prominent. Nevertheless, it is just the tip of an iceberg compared with the astronomical impact on laypeople.

Social media, such as Facebook, Twitter and Reddit, and messaging applications can facilitate rapid dissemination of new information, whether good or bad, and thus, they are the centre of infodemic warfare. For example, a Reddit^(6)^ section that discussed this paper has involved 330 comments and generated 857 upvotes, which reflects positive response on this article discussion. Then, through Twitter^(7)^ search, we have found that this article has been tweeted 1552 times and retweeted 875 times starting from 27 April, with the latest tweet on 29 June.

Furthermore, this finding has been reported in major news outlets including the *Daily Mail*^(8)^ and the *Sun*^(9)^. A news section of the British Heart Foundation^(10)^, ‘Coronavirus: Which news stories can you trust’ also discussed this preprint paper. This responsible medium provided a disclaimer that the study was a preprint and yet to be peer reviewed, while also revealing a lack of information relating to all researchers. However, other news outlets or social media users may not follow suit.

A fraudulent publication for public consumption may spread faster and overwhelm the measures to contain it. Nevertheless, preprint papers do not necessarily mean a ‘not to be trusted’ source, since various studies conducted by prominent researchers and collaborations, or NIH (National Institute of Health)-funded publications may supply us with critical information for patient care. An example of this is the RECOVERY (Randomised Evaluation of COVID-19 Therapy) Trial which enables us to provide the best treatment that will be otherwise delayed by peer reviews. A more critical approach is needed when reading preprint articles.

The authors of the current paper are from Indonesia. We launched an independent investigation to look for the Raharusuna *et al*. track record. First, we performed a search in Google Scholar, Scopus and PubMed for any prior publications by the authors. We found no records concerning the authors. Second, we performed a search in the Indonesian Medical Doctor Council Database^(11)^ and found none of the authors. Third, we searched using the Google Search Engine with keywords ‘Rumah Sakit Umum Daerah Kabupaten Sukamara’ AND ‘Prabowo Raharusuna’; we did not find any related content. Also, we performed a similar search method for ‘Prabowo Raharusun’. On searching the Indonesian Medical Council database, we ran a second search with the keyword ‘Prabowo’ and a third search with the keyword ‘Raharusun’ and found no potential author for the aforementioned preprint paper.

Since we could not find the authors’ profiles, we assessed the preprint manuscript for validity and found several issues: (1) the authors did not mention specifically the name of the hospitals (or the number of hospitals) and how they obtained the confidential data for their manuscript (similar to the Surgisphere incident). At the time this paper was written, there were only two cases of confirmed COVID-19 (both survived) in Sukamara Regency^(12)^, where the Sukamara’s Regional Public Hospital is located. Currently, the hospital is not a regional referral centre for COVID-19 care. None of the authors is affiliated to the Ministry of Health/other governmental hospitals, and they did not acknowledge any of them. (2) The name of the ethical/institutional review board was not mentioned, and there is no ethical clearance for the study. (3) Vitamin D is not routinely checked in Indonesia; the data collection method was retrospective, which is suspicious. Finally, we called the COVID-19 administration for Sukamara’s Regional Public Hospital, which is allegedly affiliated with the authors. Upon confirmation, the administrator told us that no authors’ names that we mentioned worked in this hospital. The hospital has released an official statement which can be accessed at the official website of Sukamara Regency Government^(13)^. (However, at the time the link was provided, it could only be accessed using an Indonesian network or Indonesian proxy server. Please use an Indonesian proxy server to access the page and document.)

In conclusion, we have taken several steps to investigate the identity and existence of the authors to no avail. As of the time this article was written (1 July 2020), a link to the Raharusuna *et al.* preprint at SSRN Electronic Journal cannot be accessed. However, the misinformation has been spread through various media and cited by several publications and believed by many to be true.
